# Molecular investigation of coronavirus and *Leptospira* spp. in bats (Chiroptera, Mammalia) from Brazil

**DOI:** 10.1007/s11259-026-11496-7

**Published:** 2026-09-05

**Authors:** Raquel Cuba Gaspar, Benedito Donizete Menozzi, Anna Luisa Pizzaia Henrique, Gismelli Cristiane Angeluci, Nycolas Octavio Ribeiro Carvalho, Katia Jaggi, Gabrielle dos Santos Rocha, Tayná Giovana Alaniz, Luiz Gustavo Bentim Góes, Márcia Marinho, Helio Langoni, Felipe Fornazari

**Affiliations:** 1 Department of Animal Production and Preventive Veterinary Medicine, São Paulo State University, Botucatu, São Paulo Brazil; 2 Institut Pasteur of São Paulo, São Paulo, Brazil; 3 Department of Production and Animal Health, São Paulo State University, Araçatuba, Brazil

**Keywords:** Chiroptera, Coronavirus, Leptospira, Molecular surveillance, bat-borne pathogens

## Abstract

The emergence of zoonotic diseases highlights the need for continuous surveillance of wildlife, particularly bats, known as natural reservoirs of several pathogens. This study aimed to investigate the presence of coronaviruses (CoV) and *Leptospira* spp. in non-hematophagous bats from the northwestern region of São Paulo State, Brazil, between 2019 and 2024. Samples of intestine and kidney from 98 specimens from 18 species belonging to three families (Molossidae, Phyllostomidae, and Vespertilionidae) were collected in 8 municipalities of the Araçatuba Intermediate Geographic Region. Coronaviruses detection was performed by pan-CoV nested RT-PCR, targeting fragments of 602 and 440 bp of the RNA-dependent RNA polymerase (RdRp) gene. Investigation of *Leptospira* spp. was conducted by real-time PCR, aiming the amplification of a fragment of 331 bp of the 16 S rRNA gene. The analyses yielded no amplification in any of the intestinal and renal samples. Due to geographic bias and the underrepresentation of phyllostomids – a central taxon in CoV epidemiology in Brazil -, the lack of detection reflects sampling limitations rather than the real absence of these pathogens in the local fauna. Continuous surveillance, integrated with comprehensive and standardized sampling in this under-studied region, remains essential for the early detection of emerging zoonotic pathogens and for characterizing local host-pathogen ecology.

**Supplementary Information:**

The online version contains supplementary material available at https://doi.org/10.1007/s11259-026-11496-7.

## Introduction

Bats (Chiroptera) are the second most diverse order of mammals, with about 1,500 species spread across all continents except Antarctica (Simmons and Cirranello 2025). Due to their capacity for powered flight, high metabolism, distinctive physiological traits, social behavior, wide trophic amplitude and multiple ecological functions, bats serve as a unique reservoirs for an extensive range of pathogens with zoonotic potential (Kasso and Balakrishnan 2013; Moratelli and Calisher 2015).

Research on bat-borne pathogens frequently focuses heavily on viruses (Moratelli and Calisher 2015). Among them, coronaviruses (CoV) have recently gained prominence due to the increase in human-to-human transmission, leading to public health emergencies (Weber and Silva 2024). The genera *Alphacoronavirus* and *Betacoronavirus* of the subfamily Orthocoronavirinae are associated with mammals and have clinical significance in humans (Liu et al. 2021; Woo et al. 2023).

In Brazil, the first reported case of a bat species harboring CoV occured in the state of São Paulo (Brandão et al. 2008) and since then, the virus has been detected in at least 29 bat species (Liu et al. 2026). The prevalence ranges from 0 to 19% with a predominance of the Alphacoronavirus genus (Violet-Lozano et al. 2023; Weber and Silva 2024). Detection in bats is commonly performed by PCR targeting the RNA-dependent RNA polymerase (RdRp) gene using faecal/rectal or intestinal samples (Cohen et al. 2023).

Despite the importance of bat-borne viruses, these animals can also harbor some bacteria of public health concern, including *Leptospira* (Castelo-Branco et al. 2023; Esteves et al. 2026). To date, 29 brazilian bats have tested positive for *Leptospira* spp., with a prevalence of aproximatelly 13% (Braga and Zeppelini 2025; Ferreira et al. 2026). Most studies in Brazil reported PCR results from renal samples, suggesting that bats may contribute to the environmental maintenance of the spirochete (Braga and Zeppelini 2025).

Few recent studies have investigated these pathogens in Brazil, and the northwestern region of the State of São Paulo in particular has not been assessed for over five years (Góes et al. 2013, 2016; Bittar et al. 2020). This study aimed to perform the molecular diagnosis of CoV and *Leptospira* spp. in bat tissue samples from the northwestern region of São Paulo State, Brazil.

## Materials and methods

### Ethical statement

This study was approved by the Ethics Committee on Animal Use (CEUA) of the School of Veterinary Medicine and Animal Science, São Paulo State University (UNESP), Botucatu (protocol no. 000.485).

### Sample collection

Samples of 98 individuals of 18 bat species were obtained from eight municipalities in the state of São Paulo between 2019 and 2024, as a result of rabies passive surveillance efforts. Brain smears of all these animals were analysed by direct immunofluorescence antibody test (IFAT) (WHO 2019) and tested negative for rabies in the Faculty of Veterinary Medicine of Araçatuba (FMVA/UNESP). Species were identified according to taxonomic keys to Brazilian Chiroptera (Reis et al. 2017). Tissue samples (intestine and kidney) were collected using sterile forceps and scissors and stored individually in RNase/DNase-free cryotubes (Corning^®^, USA) at − 80 °C until processing. Due to advanced autolysis, intestinal tissues from one specimen and renal tissues from four specimens could not be collected for analysis.

### CoV detection

For nucleic acids extraction, 30 mg of each intestinal tissue sample was sectioned using sterile disposable scalpel and placed into safe-lock microtubes containing 500 µL of sterile phospate-buffered saline (PBS) and three glass beads. Homogenization was performed in a TissueLyser III (Qiagen) at 30 Hz for 3 min, followed by centrifugation at 10,000 x g for 5 min. Then, 300 µL of the supernatant was loaded into the extraction plate. Automated extraction was carried out using a KingFisher Flex System (Thermo Fisher Scientific) with the Extracta^®^ DNA and RNA MDx kit (Loccus Biotecnologia), following the manufacturer’s protocol (60 µL final elution). A SARS-CoV-2-positive nasal swab and an in vitro transcript of the human coronavirus 229E target sequence were included as positive controls in each extraction batch. All isolation products were quantified using NanoDrop™ 2000 Spectrophotometer (Thermo Fisher Scientific) with a sample volume of 1 µL. Reverse transcription was performed with the High-Capacity cDNA Reverse Transcription Kit (Applied Biosystems). CoV detection was carried out by pan-coronavirus nested RT-PCR targeting two fragments of 602 and 440 bp corresponding to the RdRp gene, following the protocol described by Chu et al. (2011). Amplicons were visualized by electrophoresis on 1.5% agarose gel with addition of Unisafe DYE (20,000x) (Uniscience), ran at 145 volts for 75 min, with a 10 uL sample load and 6 uL Ladder GeneRuler 100 bp (ThermoFisher).

### *Leptospira* spp. detection

The entire kidney was minced using a sterile scalpel and tray. Genomic DNA was extracted using the ReliaPrep™ gDNA Tissue Miniprep System (Promega) according to manufacturer’s instructions and quantified following the method applied for RNA. Extracted DNA was tested in duplicate by real-time PCR (qPCR) targeting the *16 S rRNA* gene, using the primers described by Mérien et al. (1992) and SYBR Green-based chemistry, amplifying a 331 bp fragment. Reactions were performed in DNAse/RNAse-free 96-well microplates with a final volume of 20 µL, containing 1x Power SYBR^®^ Green PCR Master Mix (Thermo Fisher Scientific), 0.2 µM of each primer, 7.2 µL of nuclease-free water and 2 µL of DNA template. Nuclease-free water was used as a negative control, while gDNA extracted from the kidney tissue of a naturally infected *Rattus norvegicus* served as the positive control. Amplification results were evaluated based on cycle threshold (Ct) values and melt curve analysis (melting temperature, Tm) in comparison with the positive control.

### Data analysis

Absolute and relative frequencies were calculated for species, sex, age group, municipality of origin, and collection year, and compared descriptively. Geographic coordinates of sample origin were plotted in QGIS (v.3.40.4) using shapefiles obtained from the Brazilian Institute of Geography and Statistics (IBGE).

## Results

Ninety-eight bat specimens belonging to 18 species from three families were analyzed (Table 1). Molossidae was the most represented family, accounting for 85.7% (84/98) of individuals. Phyllostomidae contributed 8.2% (8/98) of individuals across seven species, while Vespertilionidae was the least represented family, with 6.1% (6/98) of individuals across four species. Considering all three families, 66.7% (12/18) of the identified species were represented by a single individual, indicating an asymmetric sampling distribution. Males predominated overall, totaling 64.3% (63/98) against 35.7% (35/98) females. Adults represented 93.9% (95/98) of the sample.

**Table 1:** Geographic distribution and abundance of bat species sampled in northwestern São Paulo State, Brazil, 2019–2024

Família	Espécie	Andradina	Araçatuba	Birigui	Guaraçaí	Guararapes	Ilha Solteira	Penápolis	Valparaíso	*N*	%
Molossidae	^*a*^ *Cynomops abrasus*		1							1	1
	^*b*^ *Eumops auripendulus*		1							1	1
	^*a*^ *Eumops glaucinus*		17			1	2	2		22	22.5
	*Eumops perotis*	1								1	1
	*Molossops temminckii*		1							1	1
	^*a, b*^ *Molossus molossus*		19	5		2		1		27	27.6
	^*a, b*^ *Molossus rufus*	4	14	3	1		4	5		31	31.6
Phyllostomidae	^*a*^ *Anoura caudifer*		1							1	1
	^*a, b*^ *Artibeus lituratus*		1							1	1
	^*a, b*^ *Artibeus obscurus*		1							1	1
	^*a, b*^ *Artibeus planirostris*			1						1	1
	^*a, b*^ *Glossophaga soricina*		1							1	1
	^*a*^ *Phyllostomus discolor*		2							2	2
	*Platyrrhinus infuscus*		1							1	1
Vespertilionidae	^*b*^ *Eptesicus diminutus*								1	1	1
	*Eptesicus furinalis*	1	1							2	2
	^*b*^ *Lasiurus ega*		1			1				2	2
	^*a, b*^ *Myotis nigricans*					1				1	1
		6	62	9	1	5	6	8	1	98	100

^a^. Reported with CoV (Liu et al. 2026).

^b**.**^ Reported with *Leptospira* spp. (Braga and Zeppelini 2025).

**Fig. 1:**
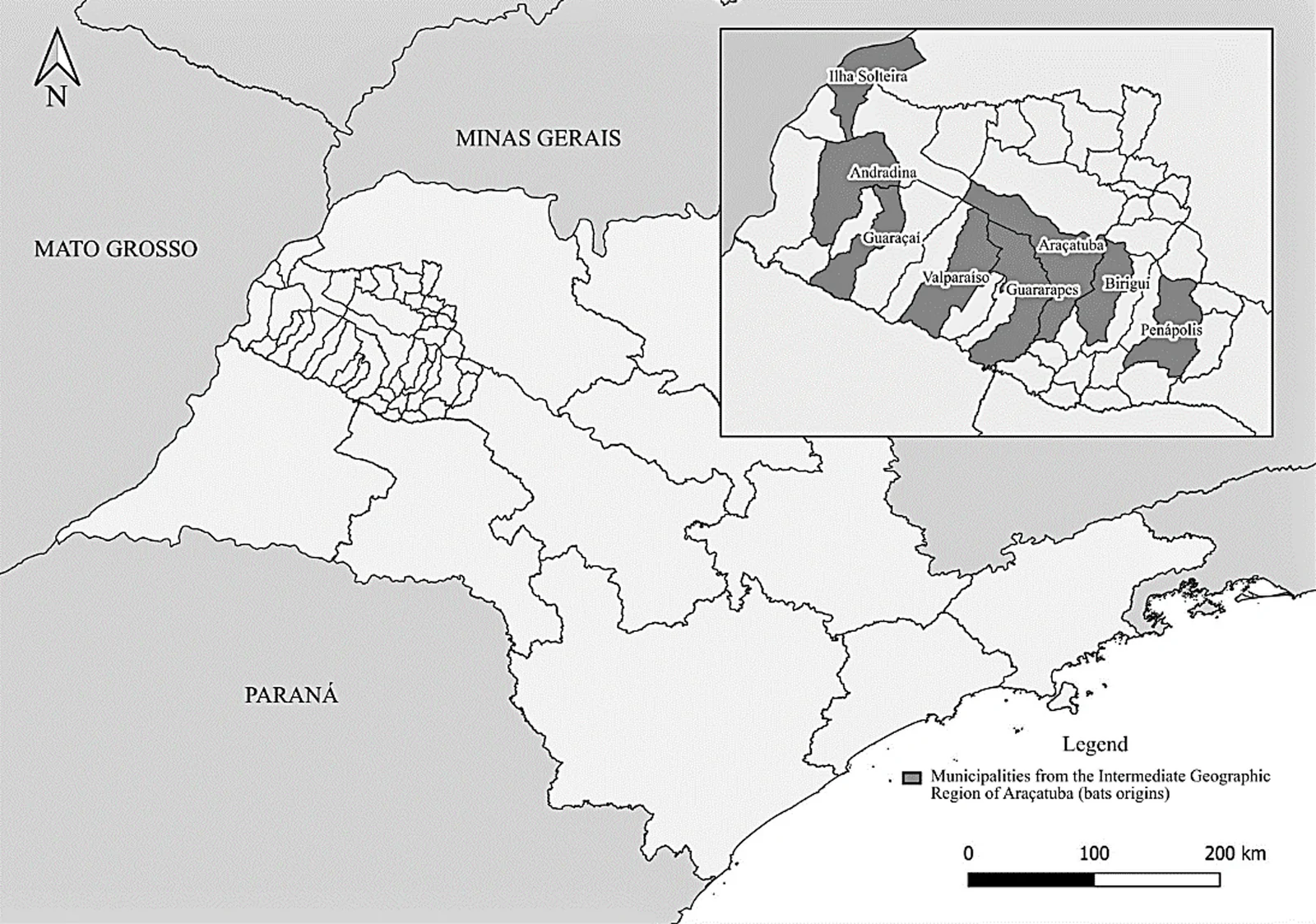
Municipalities of origin of bats - northwest region of São Paulo state, Brazil

Samples originated from eight municipalities within the Intermediate Geographic Region of Araçatuba (Fig. 1). Distribution was uneven, with Araçatuba concentrating 63.3% (62/98) of samples and was the only municipality with representatives of all three families, including all Phyllostomidae specimens except for one Artibeus planirostris from Birigui.

**Fig. 2:**
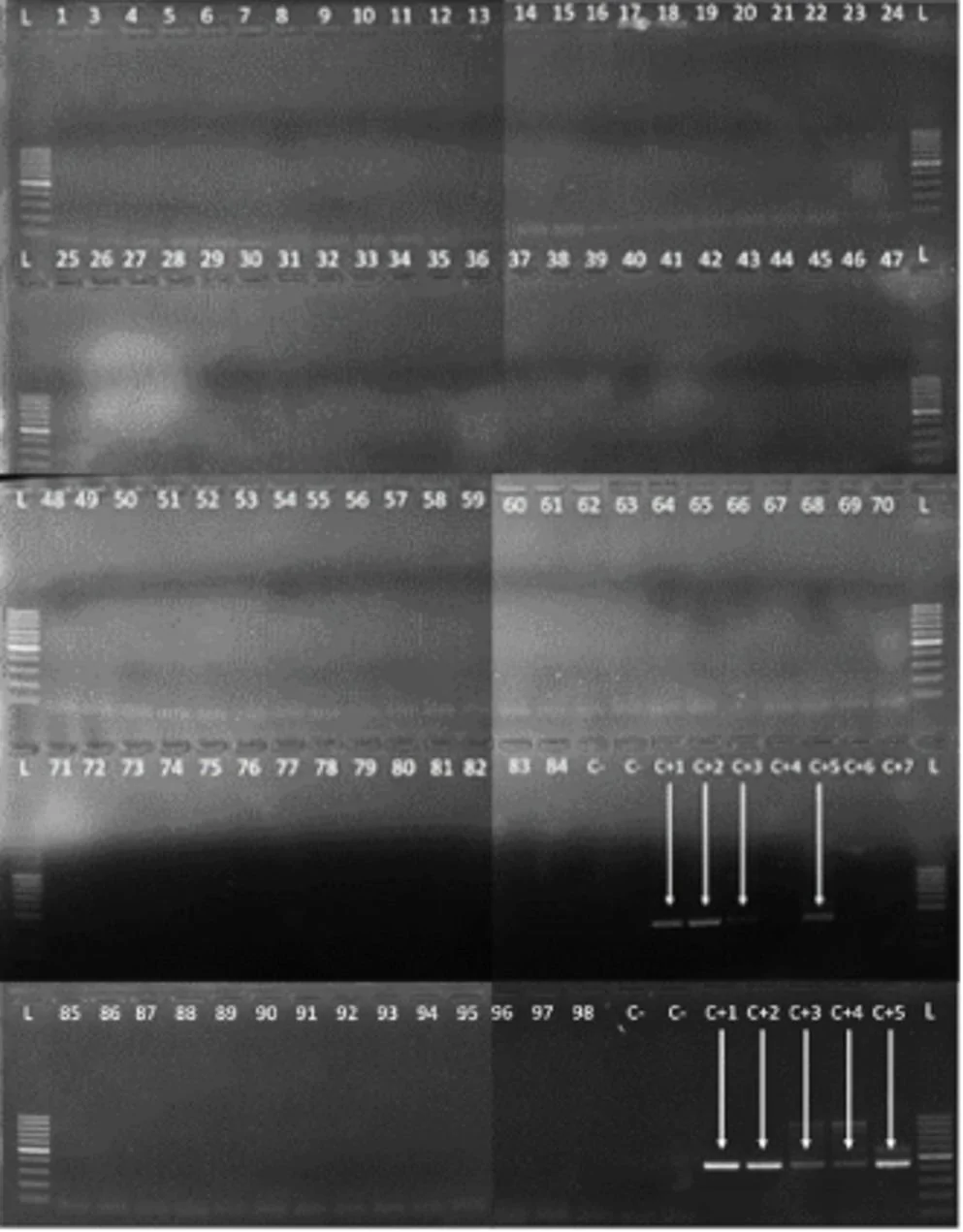
Nested reverse transcription polymerase chain reaction (RT-PCR) results for coronavirus in brazilian bats, visualized using a 1.5% agarose gel. Legend – L: ladder; C-: negative control; C + 1: HCoV229E 7 in vitro transcript; C + 2: HCoV229E 6 in vitro transcript; C + 3: SARS-CoV-2 Botucatu; C + 4: SARS-CoV-2 IPSP; C + 5: Bov Kakegawa IPSP

Regarding CoV, 11 of the 18 identified species (61.1%), representing 90.8% (89/98) of individuals, have previous CoV infection records in Brazil. Nonetheless, no amplification was observed in any of the 97 intestinal samples processed by nested RT-PCR, while positive controls performed adequately, validating the assay (Fig. 2).

**Fig. 3:**
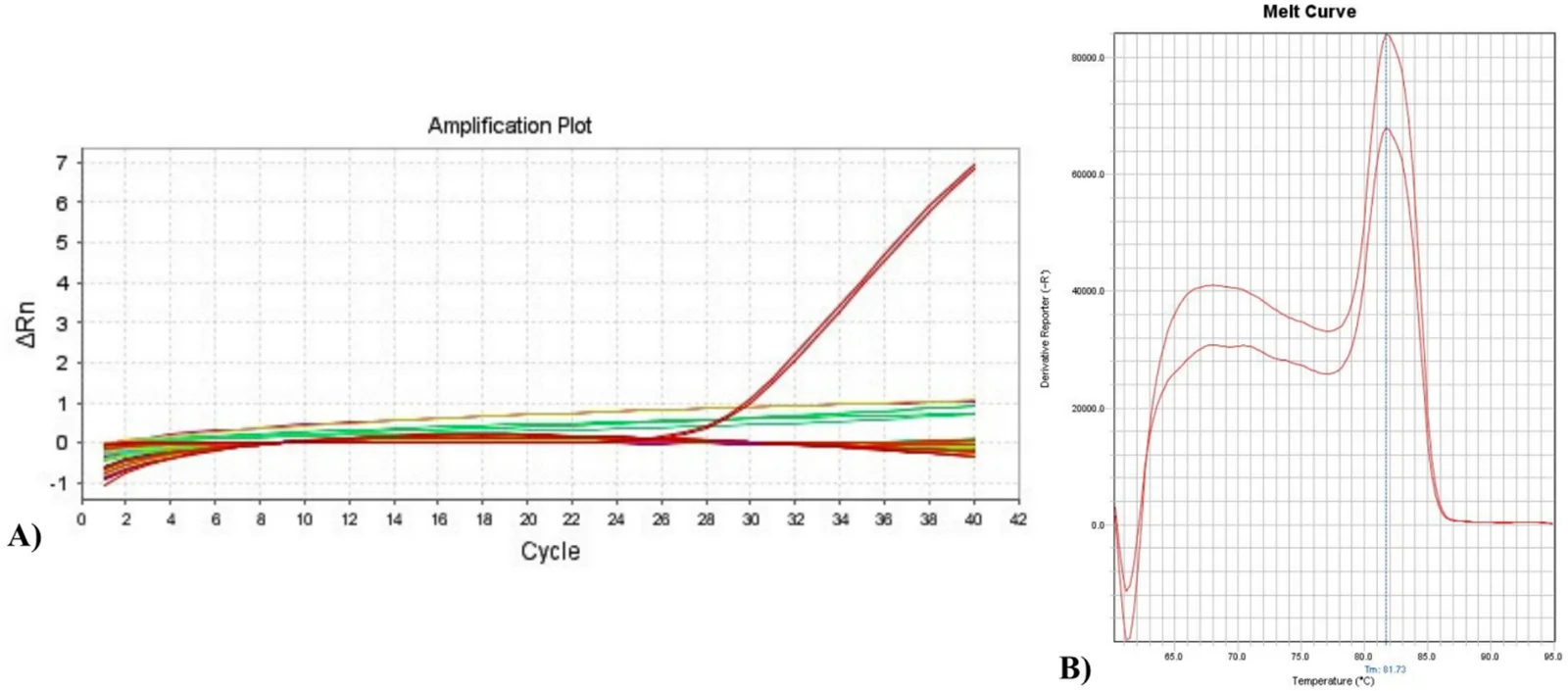
Real time polymerase chain reaction (qPCR) results for *Leptospira* spp. performed in brazilian bats. **A**) Amplification curves for the samples and positive and negative controls; **B**) dissociation curves for positive controls.

Regarding *Leptospira* spp., 12 of the 18 species (66.7%), representing 84.7% (83/98) of individuals, have previous infection records in Brazilian bats. All renal tissue samples tested negative by qPCR. Positive controls amplified as expected (Fig. 3).

## Discussion

The Chiroptera fauna found here represent 14.2% (18/127) of brazilian bats from the Atlantic Forest, the predominant biome in the Araçatuba Intermediate Geographic Region and the second richest in bat diversity in Brazil (Abreu et al. 2025). The marked dominance of Molossidae (85.7%) corroborates the sampling profile for passive surveillance studies in urban environments not only in São Paulo State (Violet-Lozano et al. 2023) but throughout Brazil (Nunes et al. 2017). This trend highlights the ecological adaptability of aerial insectivorous species which commonly use buildings as roosts and exploit food availability around public lighting (Nunes et al. 2017, Jung and Threlfall 2018).

Conversely, the low representation of Phyllostomidae suggests scarcity of resources limiting the presence of pollinators and seed dispersers in the region, since they usually carry out their activities in native forest remnants (Nunes et al. 2017) and rarely use artificial structures as shelter (Faria 2006). This scenario contrasts with active mist-net studies (Cosentino et al. 2026). The under-sampling of Phyllostomidae represents an important gap for viral surveillance, since this family accounts for a large proportion of CoV records in Brazilian bats (Liu et al. 2026).

Samples concentration in Araçatuba reflects a sampling bias associated with the location of the reference laboratory, limiting definitive conclusions about regional biodiversity and pathogen ecology. This pattern, together with the high proportion of single-individual species records, suggests that the region remains under-sampled — a common situation in convenience-based CoV surveillance studies, which can be worked out with repeated, longitudinal sampling substantially increasing detection probability, whereas single sampling events — as in the present study — tend to underestimate true prevalence (Cohen et al. 2023).

Although the negative CoV result aligns with findings from a similiar study in São Paulo State (Violet-Lozano et al. 2023), thereby supporting the hypothesis of low viral prevalence within these urban populations, our data contrast with historial records from the same region. Alphacoronavirus was identified in *Molossus rufus* and *Molossus molossus* (Góes et al. 2013) — the predominant species in our casuistry — alongside reported prevalence rates of 17.18%, with systemic viral detection extending across intestinal tissue (Bittar et al. 2020). Pooled fecal sampling in urban roosts has yielded great sensitivity (prevalence of 19.3%) (Lima et al. 2013); furthermore, multi-tissue screening enhances overall detection probability, as viral replication site can vary among host species and CoV genotypes (Bittar et al. 2020).

Regarding *Leptospira* spp., the absence of amplification across all 94 renal samples should be interpreted within the broader context of national sampling constrainsts. Only eight studies on *Leptospira* in Brazilian bats were published between 1976 and 2024, spanning five of the 27 federative units (Braga and Zeppelini 2025). Despite the negative outcome, the present study extends national geographic coverage by providing unprecedented data for northwestern São Paulo State. The sampling strategy employed here differs from the active mist-net capture methods used in most Brazilian studies. Nevertheless, comparable convenience sampling from rabies diagnostic services has yielded high *Leptospira* positivity elsewhere in Brazil — such as 39.1% in Rio Grande do Sul, with emphasis on *Tadarida brasiliensis* and *Molossus molossus* (Mayer et al. 2017), and 22.5% in the same state, accompanied by phylogenetic confirmation of *Leptospira interrogans* (Ulsenheimer et al. 2022). By contrast, in southeastern small town region were found no PCR amplification in blood, despite detecting anti-*Leptospira* antibodies by microscopic agglutination in 6.1% of animals (Machado et al. 2023). This regional pattern, more aligned with our findings, supports the hypothesis of marked geographic variation in *Leptospira* circulation between São Paulo State and southern Brazil. Finally, regarding the molecular detection of *Leptospira*, it is unlikely that the PCR target used in this study affected the likelihood of detecting positive animals. Most studies conducted for diagnostic purposes employ primers targeting the 16S rRNA and LipL32 genes, both of which have demonstrated high analytical sensitivity for the detection of *Leptospira* spp.

Rather than indicating absolute absence of these agents, these negative findings underscore the methodological limitations of point-in-time passive surveillance and should by no means justify a reduction in monitoring efforts. To establish a more robust and definitive framework for coronavirus and *Leptospira* spp. ecology in this historically understudied region, is suggested that future research must incorporate expanded geographic coverage, enhanced sampling of the Phyllostomidae Family, longitudinal multi-tissue screening and complementary diagnostic techniques.

## Supplementary Information

Below is the link to the electronic supplementary material.

Supplementary Material 1 (DOCX 34.7 KB)

## Data Availability

All data supporting the findings of this study are available within the paper and its Supplementary Information.
