# Supplementary material for: Molecular investigation of coronavirus and Leptospira spp. in bats (Chiroptera, Mammalia) from Brazil
Source: Vet Res Commun. 2026 Sep 5;50(6):554. doi: 10.1007/s11259-026-11496-7 (PMC13546309; doi:10.1007/s11259-026-11496-7)
Supplement: Supplementary file 1 [file 11259_2026_11496_MOESM1_ESM.docx]

**Table 1.** Assessment of yield (ng/uL) and purity ratios (A260/280 and A260/230) of ribonucleic acid (RNA) and deoxyribonucleic acid (DNA) in tissue samples of bats from Brazil.

|  | Intestinal tissue (RNA 40) | | | Renal tissue (DNA 50) | | |
| --- | --- | --- | --- | --- | --- | --- |
| Sample | ng/uL | 260/280 | 260/230 | ng/uL | 260/280 | 260/230 |
| RF-001 | 564,70 | 2,00 | 2,13 | 113,70 | 1,91 | 2,32 |
| RF-002 | 0,00 | 0,00 | 0,00 | 0,00 | 0,00 | 0,00 |
| RF-003 | 1055,00 | 1,93 | 2,27 | 93,10 | 1,93 | 2,29 |
| RF-004 | 642,60 | 2,09 | 2,32 | 78,10 | 2,13 | 2,21 |
| RF-005 | 1870,70 | 2,04 | 2,09 | 73,90 | 2,11 | 1,95 |
| RF-006 | 937,20 | 2,00 | 2,24 | 181,70 | 2,02 | 2,27 |
| RF-007 | 246,60 | 2,42 | 2,36 | 12,00 | 2,50 | 2,07 |
| RF-008 | 633,60 | 2,07 | 2,07 | 56,00 | 2,20 | 2,13 |
| RF-009 | 336,20 | 2,22 | 2,31 | 117,80 | 2,06 | 2,14 |
| RF-010 | 281,80 | 2,28 | 1,71 | 20,10 | 2,25 | 2,07 |
| RF-011 | 125,80 | 2,86 | 1,16 | 6,70 | 2,22 | 1,13 |
| RF-012 | 389,00 | 2,09 | 2,03 | 158,50 | 1,92 | 2,25 |
| RF-013 | 140,60 | 2,79 | 1,64 | 51,30 | 2,17 | 2,01 |
| RF-014 | 292,80 | 2,28 | 2,06 | 18,20 | 2,33 | 1,96 |
| RF-015 | 211,70 | 2,47 | 2,34 | 14,30 | 2,41 | 2,07 |
| RF-016 | 317,50 | 2,20 | 2,06 | 0,00 | 0,00 | 0,00 |
| RF-017 | 430,50 | 2,07 | 2,14 | 119,60 | 1,92 | 2,39 |
| RF-018 | 323,40 | 2,19 | 2,05 | 20,60 | 2,14 | 2,82 |
| RF-019 | 546,30 | 2,10 | 2,43 | 15,50 | 2,26 | 2,06 |
| RF-020 | 325,70 | 2,22 | 2,28 | 13,30 | 2,08 | 2,08 |
| RF-021 | 303,70 | 2,16 | 1,28 | 22,70 | 2,08 | 2,33 |
| RF-022 | 1532,00 | 1,98 | 2,24 | 72,40 | 2,10 | 2,12 |
| RF-023 | 341,70 | 2,24 | 0,72 | 22,90 | 2,09 | 1,26 |
| RF-024 | 363,30 | 2,19 | 2,01 | 56,60 | 2,12 | 2,15 |
| RF-025 | 129,50 | 2,78 | 1,57 | 6,40 | 2,99 | 1,81 |
| RF-026 | 138,90 | 2,67 | 2,28 | 132,70 | 1,92 | 2,22 |
| RF-027 | 126,20 | 2,79 | 2,04 | 3,10 | 2,26 | 0,67 |
| RF-028 | 286,70 | 2,29 | 2,38 | 8,50 | 2,22 | 1,31 |
| RF-029 | 137,40 | 2,65 | 1,18 | 5,10 | 2,92 | 1,75 |
| RF-030 | 75,80 | 3,29 | 2,22 | 2,90 | 3,61 | 0,91 |
| RF-031 | 294,90 | 2,27 | 2,27 | 57,00 | 2,07 | 2,19 |
| RF-032 | 195,00 | 2,37 | 2,48 | 6,80 | 2,45 | 1,65 |
| RF-033 | 100,30 | 2,98 | 1,73 | 8,20 | 2,92 | 1,57 |
| RF-034 | 96,80 | 3,05 | 2,56 | 7,20 | 2,20 | 1,28 |
| RF-035 | 267,40 | 2,19 | 1,53 | 0,00 | 0,00 | 0,00 |
| RF-036 | 503,90 | 2,07 | 2,45 | 98,90 | 1,92 | 2,44 |
| RF-037 | 99,20 | 2,96 | 0,42 | 3,60 | 2,94 | 1,44 |
| RF-038 | 99,40 | 3,43 | 0,20 | 0,00 | 0,00 | 0,00 |
| RF-039 | 237,20 | 2,34 | 1,86 | 6,20 | 2,40 | 1,92 |
| RF-040 | 370,50 | 2,15 | 1,17 | 167,80 | 1,91 | 2,20 |
| RF-041 | 996,60 | 1,95 | 2,28 | 200,10 | 1,93 | 2,32 |
| RF-042 | 208,80 | 2,39 | 0,81 | 12,20 | 2,41 | 2,12 |
| RF-043 | 193,30 | 2,44 | 1,75 | 97,50 | 1,61 | 2,22 |
| RF-044 | 464,40 | 2,01 | 1,70 | 137,80 | 1,89 | 2,31 |
| RF-045 | 188,50 | 2,45 | 1,11 | 84,00 | 1,79 | 2,31 |
| RF-046 | 305,20 | 2,19 | 1,43 | 13,20 | 2,04 | 2,13 |
| RF-047 | 129,30 | 2,84 | 1,30 | 30,30 | 1,61 | 2,17 |
| RF-048 | 254,20 | 2,25 | 1,05 | 126,80 | 1,91 | 2,34 |
| RF-049 | 434,80 | 2,07 | 1,80 | 13,20 | 2,21 | 2,11 |
| RF-050 | 138,20 | 2,77 | 2,19 | 123,20 | 2,04 | 2,22 |
| RF-051 | 138,70 | 2,69 | 1,33 | 0,00 | 0,00 | 0,00 |
| RF-052 | 260,80 | 2,12 | 1,12 | 154,80 | 1,96 | 2,18 |
| RF-053 | 511,00 | 2,08 | 2,43 | 54,20 | 2,12 | 2,07 |
| RF-054 | 399,20 | 2,08 | 2,09 | 213,70 | 1,82 | 2,26 |
| RF-055 | 289,10 | 2,19 | 2,01 | 128,40 | 1,91 | 1,98 |
| RF-056 | 224,00 | 2,31 | 2,15 | 133,20 | 1,93 | 1,92 |
| RF-057 | 156,20 | 2,58 | 2,32 | 32,00 | 2,10 | 1,81 |
| RF-058 | 102,40 | 3,07 | 2,27 | 10,90 | 1,97 | 0,90 |
| RF-059 | 300,90 | 2,21 | 2,34 | 157,80 | 1,95 | 2,13 |
| RF-060 | 350,80 | 2,15 | 2,07 | 87,40 | 1,95 | 1,88 |
| RF-061 | 120,60 | 2,87 | 0,38 | 10,30 | 1,32 | 1,73 |
| RF-062 | 98,60 | 3,17 | 2,55 | 5,70 | 1,80 | 0,76 |
| RF-063 | 437,70 | 2,07 | 2,24 | 3,60 | 3,26 | 1,24 |
| RF-064 | 113,60 | 2,86 | 1,40 | 2,10 | 2,01 | -3,67 |
| RF-065 | 386,20 | 2,10 | 1,67 | 214,20 | 1,89 | 1,83 |
| RF-066 | 446,30 | 2,09 | 1,10 | 28,50 | 0,93 | 0,39 |
| RF-067 | 322,30 | 2,14 | 1,39 | 316,90 | 1,90 | 2,16 |
| RF-068 | 125,20 | 2,77 | 0,34 | 275,60 | 1,94 | 2,06 |
| RF-069 | 102,00 | 2,99 | 0,69 | 44,60 | 2,13 | 1,92 |
| RF-070 | 289,30 | 2,26 | 1,13 | 161,40 | 1,94 | 1,60 |
| RF-071 | 248,80 | 2,27 | 0,70 | 22,20 | 2,16 | 1,75 |
| RF-072 | 111,50 | 2,92 | 1,18 | 789,90 | 1,86 | 2,05 |
| RF-073 | 434,00 | 2,15 | 2,06 | 10,90 | 2,08 | 1,60 |
| RF-074 | 259,30 | 2,25 | 1,14 | 25,80 | 2,00 | 1,97 |
| RF-075 | 422,50 | 2,10 | 1,01 | 12,50 | 2,07 | 1,27 |
| RF-076 | 271,10 | 2,20 | 1,48 | 8,50 | 1,70 | 0,93 |
| RF-077 | 228,00 | 2,39 | 0,70 | 189,60 | 1,90 | 2,29 |
| RF-078 | 167,50 | 3,06 | 0,37 | 108,20 | 0,83 | 1,73 |
| RF-079 | 91,70 | 3,30 | 1,06 | 4,00 | 1,67 | 1,05 |
| RF-080 | 105,00 | 3,13 | 0,88 | 13,20 | 1,80 | 0,95 |
| RF-081 | 155,80 | 2,62 | 1,01 | 4,50 | 2,89 | 1,20 |
| RF-082 | 104,90 | 2,97 | 0,70 | 4,70 | 1,99 | 1,30 |
| RF-083 | 184,60 | 2,46 | 0,81 | 6,40 | 1,93 | 1,30 |
| RF-084 | 104,50 | 2,73 | 0,68 | 5,10 | 2,18 | 1,51 |
| RF-085 | 96,40 | 2,77 | 1,35 | 75,50 | 1,08 | 1,55 |
| RF-086 | 205,10 | 2,41 | 1,38 | 57,80 | 1,85 | 2,04 |
| RF-087 | 177,50 | 2,66 | 0,35 | 94,40 | 1,89 | 1,82 |
| RF-088 | 189,60 | 2,50 | 0,37 | 60,20 | 1,21 | 1,78 |
| RF-089 | 136,10 | 2,74 | 1,96 | 47,80 | 1,94 | 1,51 |
| RF-090 | 153,90 | 2,71 | 0,52 | 61,70 | 1,28 | 1,60 |
| RF-091 | 114,60 | 2,91 | 1,78 | 73,00 | 0,66 | 1,51 |
| RF-092 | 424,30 | 2,16 | 2,24 | 128,10 | 1,99 | 2,11 |
| RF-093 | 463,20 | 2,12 | 2,27 | 194,40 | 2,10 | 1,95 |
| RF-094 | 192,10 | 2,45 | 2,44 | 34,10 | 2,13 | 1,76 |
| RF-095 | 338,00 | 2,16 | 1,95 | 62,90 | 2,01 | 1,78 |
| RF-096 | 105,30 | 3,10 | 1,85 | 40,10 | 1,90 | 1,89 |
| RF-097 | 76,00 | 3,19 | 1,71 | 9,90 | 1,30 | 0,35 |
| RF-098 | 252,90 | 2,22 | 1,96 | 249,90 | 1,84 | 1,72 |
| Mean | 302,79 | 2,43 | 1,62 | 72,68 | 1,92 | 1,66 |
